# Hyperlipidemia-induced metabolic dysregulation impairs tendon homeostasis: the role of TGF-*β*/Smad2 signaling pathway in lipid-mediated extracellular matrix remodeling

**DOI:** 10.3389/fmed.2026.1820393

**Published:** 2026-04-17

**Authors:** Peiyang Shang, Renxuan Li, Yan Xia, Zhen Wang, Renhao Yang, Qingge Fu

**Affiliations:** 1 Department of Orthopaedics, Putuo Hospital, Shanghai University of Traditional Chinese Medicine, Shanghai, China; 2 Shanghai Key Laboratory for Prevention and Treatment of Bone and Joint Diseases, Department of Orthopedics, Sports Medicine Center, Shanghai Institute of Traumatology and Orthopaedics, Ruijin Hospital, Shanghai Jiao Tong University School of Medicine, Shanghai, China

**Keywords:** ECM remodeling, hyperlipidemia, ox-LDL, tendon homeostasis, TGF-*β*/Smad2 signaling

## Abstract

**Background:**

Hyperlipidemia is a globally prevalent metabolic disorder, and the lipid metabolic dysregulation it induces is closely associated with the onset and progression of tendon pathologies, which are typically characterized by ECM dyshomeostasis, disorganized collagen fiber structure and impaired biomechanical properties in the affected tendons. The TGF-*β* signaling pathway serves as a central regulator of tendon ECM metabolic homeostasis, among which the TGF-β/Smad2 axis can directly mediate the balanced regulation of collagen synthesis and degradation in tendons, yet its specific role and underlying molecular mechanism in hyperlipidemia-induced tendon ECM dysfunction remain poorly defined.

**Methods:**

We examined how oxidized lipids affect the TGF-*β*/Smad2 signaling axis in tendon tissue. Specifically, we assessed TβRII localization within lipid rafts, TGF-β activation, downstream transcription of Col1a1 and lysyl oxidase (LOX), and matrix metalloproteinase-1 (MMP-1) activity.

**Results:**

Oxidized lipids disrupted T*β*RII localization in lipid rafts, leading to impaired TGF-β activation. This suppressed Smad2 signaling, decreased Col1a1 transcription and LOX expression, and increased MMP-1 activity. Consequently, tendon collagen synthesis declined while degradation rose, undermining structural integrity and intrinsic repair capacity.

**Conclusion:**

Hyperlipidemia-driven suppression of the TGF-*β*/Smad2 axis is the critical mechanism linking metabolic dysregulation to tendon matrix failure. Restoring TGF-β/Smad2 signaling represents a promising therapeutic strategy to mitigate hyperlipidemia-induced tendon damage and poor healing.

## 1 Introduction

Hyperlipidemia, a prevalent metabolic disorder, has seen a rising incidence in recent years due to the change of the dietary patterns, occupational stress, and lifestyle changes (1–3). This hyperlipidemic condition is marked by significant elevations in plasma concentrations of triglycerides (TG), total cholesterol (TC), and low-density lipoprotein cholesterol (LDL-C), alongside reduced high-density lipoprotein cholesterol (HDL-C) (4). Epidemiological evidence highlights a significant correlation between hyperlipidemia and tendon pathologies (e.g., tendinitis and spontaneous rupture), with affected tissues exhibiting lipid infiltration, collagen disorganization, and impaired biomechanical properties (5). Although chronic inflammation and oxidative stress are proposed as potential mechanisms for lipid-induced tendon homeostasis disorder, the molecular link between metabolic dysregulation and extracellular matrix (ECM) remodeling remains poorly defined.

As the primary mechanical transducer, tendon is a dense connective tissue predominantly composed of hierarchically organized type I collagen fibers and its force-transmitting capacity relies on their highly ordered hierarchical structure (6). As for normal tendon homeostasis, type I collagen constitutes over 95% of total collagen in tendon tissue, forming a unique load-adaptive structure through multiscale assembly (7). However, the key signaling pathways driving hyperlipidemia-induced alterations in tendon structural integrity remain mechanistically unclear. Tenocyte is the principal resident cells in tendons, maintain ECM homeostasis through matrix synthesis (8). Nevertheless, low cellularity and hypovascularity in tendon tissue result in low metabolic activity and limited adaptability, rendering tendons vulnerable to persistent homeostasis insults (9). Some studies indicate that hyperlipidemic conditions promote ectopic lipid deposition and ultrastructural tendon damage (10). However, prior research has predominantly focused on secondary inflammatory cascades or mechanical overload theories, overlooking the direct regulatory effects of lipid metabolites on tenocyte signaling networks. This knowledge gap has obscured the causal relationship between metabolic dysfunction and collagen synthesis failure.

The TGF-*β* signaling pathway serves as a central regulator of tendon metabolic homeostasis, balancing collagen synthesis/degradation and integrating mechanical/metabolic cues to preserve structural and functional integrity (11, 12). Under hyperlipidemia, oxidized low-density lipoprotein (OxLDL) disrupts T*β*RII localization within lipid rafts, inhibiting TGF-β signaling activation (13). This leads to the reduction in COL1A1 transcriptional activity and downregulation of LOX expression, but increase in MMP-1 activity, which would lead to collagen synthesis insufficiency and excessive degradation (14, 15). Nevertheless, the precise mechanisms underlying TGF-*β*-mediated ECM dysregulation in this context remain unresolved. Identifying and modulating TGF-*β*-associated pathways governing collagen synthesis is thus critical for reversing hyperlipidemia-induced tendon ECM damage.

Within TGF-β signaling, the TGF-β/Smad2 axis directly regulates expression of key ECM components (e.g., type I/III collagen, fibronectin) and suppresses matrix metalloproteinases (MMP-1, MMP-3) in tendon homeostasis (16). However, its role in hyperlipidemia-associated tendon metabolic imbalance has not been investigated. This study reveals that the TGF-*β*/Smad2 axis can influence both native tendon architecture and post-injury collagen deposition/alignment. These findings elucidate the molecular mechanisms of hyperlipidemia-driven tendon pathophysiology and identify translational therapeutic targets for clinical intervention.

## 2 Materials and methods

### 2.1 TEM scanning for tendon tissue

The ApoE −/− mice (purchased from GemPharmatech Co., Ltd) and wild-type (WT) littermates (purchased from GemPharmatech Co., Ltd) were housed under specific pathogen-free conditions with ad libitum access to standard chow and water. Achilles and patellar tendons were rapidly dissected and fixed in ice-cold 4% paraformaldehyde/2.5% glutaraldehyde in 0.1 M phosphate buffer (pH 7.4) for 24 h and at 4 °C. For transmission electron microscopy (TEM), tissues were post-fixed in 1% osmium tetroxide with 1.5% potassium ferrocyanide, dehydrated in graded ethanol, and embedded in Epon 812 resin. Ultrathin sections (70 nm) were stained with uranyl acetate and lead citrate, then imaged using a Hitachi HT7800 TEM (80 kV). We observed collagen fibers, cell morphology and mitochondrial morphology in tendons.

### 2.2 Tenocyte culture

Isolating the tendon tissue from the Achilles tendons 8-week-old Sprague–Dawley rats and cutting the tissue into 5 mm^3^ then placing the pieces into the 100-mm culture plates, for which primary tenocytes migrated out of the tendon pieces and started expanding at the bottom of the culture plates. The plates maintaines in DMEM with 10% FBS (Gibco) at 37 °C in a humidified atmosphere of 5% CO_2_ and the medium was changed every 3 days. Finally, when the concentration of tenocytes reached 70–80% at about day 10–14, the cells were trypsinized and subcultured onto 100-mm dishes.

### 2.3 Cell viability

The CCK8 (Cell Counting Kit-8) assay was used to assess tenocyte viability. Tenocytes were seeded in 96-well plates and then incubated in 25, 50 and 100 μg/mL OxLDL medium. After culturing for 3 days, medium was replaced with DMEM medium containing 10% CCK8 reagents, and the plates were incubated at 37 °C for 1 h. Afterwards, the plates were absorbed at 450 nm were detected using an ELISA reader (Multiskan FC, Thermo Fisher).

### 2.4 Live/dead staining

After 48 h of incubation and 25, 50 and 100 μg/mL OxLDL medium stimulation, cells were stained with the Live/Dead™ Kit for 30 min. Fluorescence microscopy (ZEISS, Axio Imager M1, Germany) was then used for observation.

### 2.5 Cell apoptosis

The cells were harvested, washed twice with ice-cold PBS, and processed for dual analyses. For cell cycle profiling, cells were stained with propidium iodide (PI, 50 μg/mL; Thermo Fisher) for 30 min in the dark. All samples were analyzed immediately on the flow cytometer (FACSCanto II, BD Biosciences), acquiring ≥10,000 viable single-cell events per replicate.

### 2.6 Real-time quantitative PCR analysis

Total RNA was extracted from cells on 6-well plates using TRI Reagent and Direct-zolTM RNA Kit (Zymo Research), and 500 ng RNA was reverse transcribed into cDNA with Tetro cDNA Synthesis Kit (Bioline). Quantitative real-time PCR was performed using the ABI 7900 system (Applied Biosystems) with a SensiFASTTM SYBR® Hi-ROX Kit (Bioline), where each reaction mixture contained 50 ng of cDNA. Sequences of the primer pairs used were:

GAPDH, forward primer (5′-GTGGACCTCATGGCCTACAT-3′),

reverse primer (5′-GGATGGAATTGTGAGGGAGA-3′);

Scleraxis (Scx), forward primer (5′-TTGAGCAAAGACCGTGACAG-3′),

reverse primer (5′-CTGTGCTCAGATCAGGTCCA-3′);

Col1a1, forward primer (5′-GGAAGAGCGGAGAGTACTGG-3′),

reverse primer (5′-CATGCTCTCTCCAAACCAGA-3′);

Smad2, forward primer (5′-CAGCCATCATCTTCGACAGC-3′),

reverse primer (5′-GGTGGCATAGTCCTGGTTGT-3′).

The quantitative real-time PCR (qPCR) was performed using a three-step cycling protocol: initial polymerase activation at 95 °C for 2 min, followed by 40 cycles of denaturation (95 °C, 5 s), primer annealing (60 °C, 10 s), and elongation (72 °C, 10 s). Specificity of amplified products was confirmed by melting curve analysis with gradual temperature ramping from 60 °C to 95 °C. Gene expression quantification employed the comparative 2(−ΔΔCT) method, with GAPDH as internal controls.

### 2.7 Western blotting

Total soluble protein was collected from cells on 6-well plates using RIPA buffer. The SDS-PAGE was performed with a 10% gel, and then the proteins were transferred onto PVDF membranes. Following blocking of the membrane by TBST buffer containing 0.05% Tween 20 and 5% BSA for 1.5 h at room temperature, the membrane was incubated in the buffer with primary antibody targeting phospho-Smad2, Smad2, Col1a1, Scx or GAPDH at 4 °C overnight and then with HRP-conjugated secondary antibody. After adding Chemiluminescent HRP Substrate to the membrane, the luminescence images were acquired by ImageQuant LAS 4000 (GE Healthcare, Uppsala, Sweden).

### 2.8 *In vivo* experiment

Mice Achilles tendon acute injury model: The animal experiment was approved by the Animal Ethics Commitee of Putuo Distriet Center Hospital, Shanghai (DWEC-A-2025-27-1-125). The ApoE^−/−^ and normal mice were used as animal models and the hindlimbs were sterilized. The tendon repair model was carried out by repairing the damaged Achilles tendon using a modified Kessler technique and 6–0 nonabsorbable sutures (Ethicon Ltd., Edinburgh, UK) (17). The animals were taken at 4 and 8 weeks.

### 2.9 Histologic evaluation

Separating the Achilles tendon tissue at 4 and 8 weeks after surgery and fixed in paraformaldehyde for 24 h and wrapped and cut into 5-μm-thick sagittal and cross-section slices. Then the hematoxylin and eosin (H&E) staining and picrosirius red staining were performed and observed under a light and polarized light microscope (Germany, Leica). The Sirius red staining was used to observe and assess the distribution and content of type I and III collagen in the total repaired tissue.

### 2.10 Biomechanical evaluation

The biological strength of the repaired tendon was evaluated with a longitudinal load. The device was pulled at a constant speed of 1 mm/min, the tensile load (N) was gradually increased until the tendon ruptured from the suture, and the results were entered into TRAPEZIUM X 13.0 mechanical analysis software.

### 2.11 Statistical analysis

All the data are expressed as mean ± SEM. To evaluate the differences between two groups, an independent t-test was performed, and for comparisons between three or four groups, one-way ANOVA was performed. Tukey’s *post hoc* test was used to examine the glucose effect or insulin effect, following a significant ANOVA. Differences with a *p*-value less than 0.05 were significant.

## 3 Results

### 3.1 Effects of hyperlipidemia on tendon tissue

**Figure 1:**
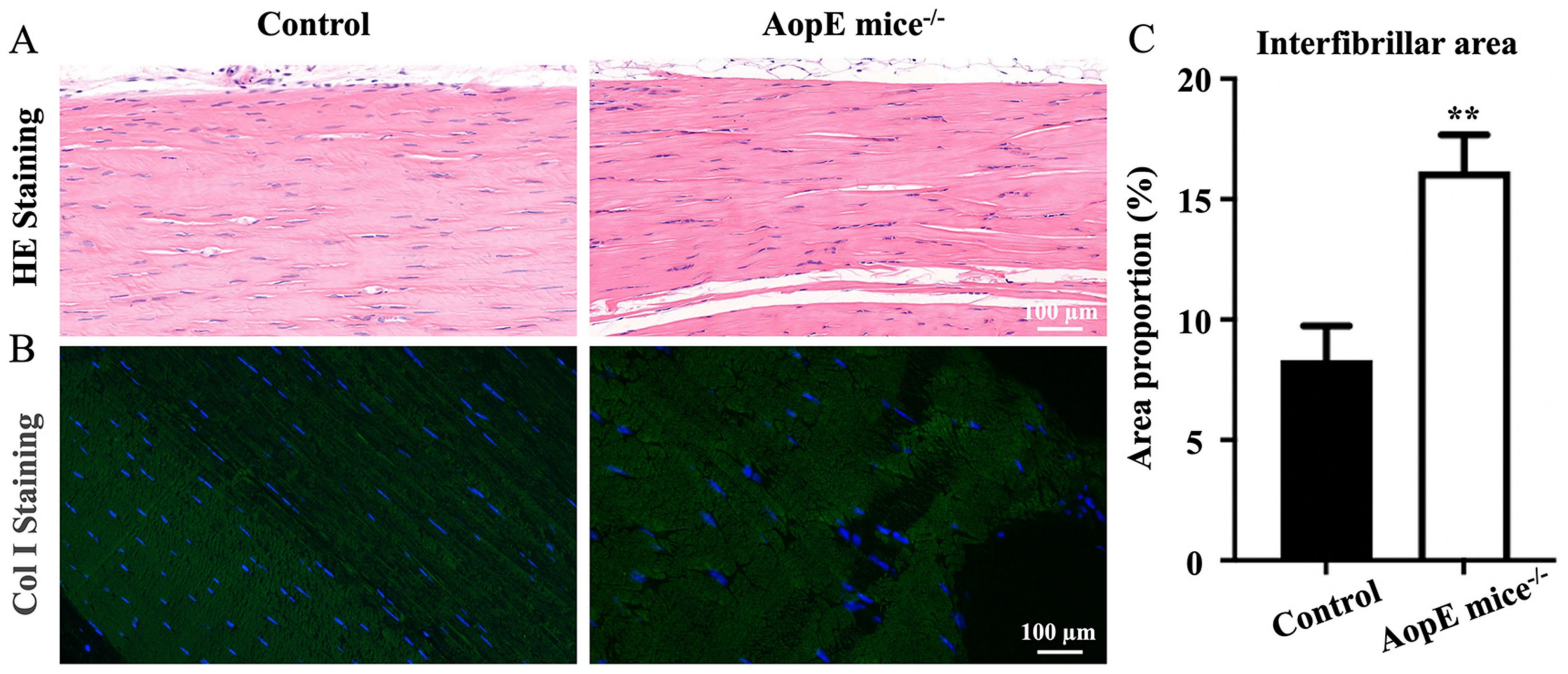
Changes in tendon morphology in hyperlipidemia. **(A)** H&E staining results of control (normal) and ApoE^−/−^ mice; **(B)** collagen I immunofluorescence staining results of control and ApoE^−/−^ group; **(C)** interfibrillar areas based on H&E staining results and shown as percentages (*n* = 3; **p < 0.01 compared with control group).

To determine how tendon tissues would alter after long-term hyperlipidemia, we dissected the Achilles tendons from ApoE−/− mice and analyzed the sections via hematoxylin and eosin (H&E) and immunofluorescence staining. The H&E staining results indicated that collagen fibers were more loosely arranged and showed weaker fiber tension in the tendon tissue from ApoE−/− mice (Figure 1A). The immunofluorescence staining results found that the brightness of type I collagen was higher indicating higher content and the distribution of type I collagen showed denser and more orderly (Figure 1B). Through above histological analysis, we confirmed that the average interfibrillar area of tendon was significantly larger in ApoE−/− mice than in normal mice (Figure 1C).

### 3.2 Hyperlipidemia altered microstructure and related protein expression of tendon

**Figure 2:**
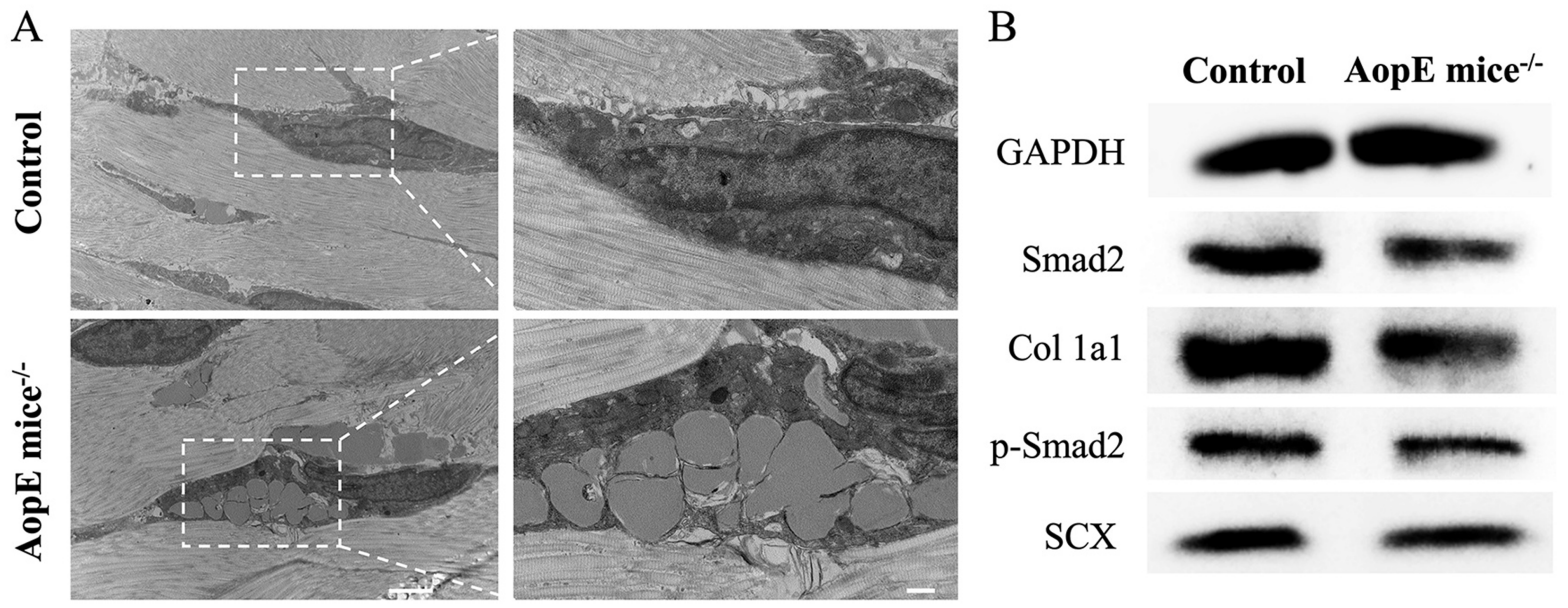
Alterations in microstructure and protein expression in hyperlipidemia. **(A)** The TEM results of the tendon tissue from the control (normal) and *ApoE*^−/−^ mice; **(B)** Related western blotting results of tendon tissue in different group.

First, gross morphological observation of the tendons was conducted. Compared with the control group, tendons from ApoE−/− mice exhibited a rough surface contour and indistinct margins, lacking the regular morphology characteristic of normal tendons (Supplementary Figure S1). Furthermore, we used TEM to detect the ultrastructure of tissues and cells in tendon tissue and found that there were marked expansion of the interfibrillar gaps between collagen fibrils and prominent intracellular accumulation of lipidic inclusions in tenocytes in the ApoE^−/−^ group, proving that hyperlipidemia directly affects the microstructure of cells and the surrounding extracellular matrix (Figure 2A). To further test our hypothesis, we performed western blotting analysis and found that Smad2, p-Smad2, Col 1a1 and SCX expressed less in the ApoE^−/−^ group than in the control group (Figure 2B and Supplementary Figure S2).

### 3.3 The effects of OxLDL on tenocytes

**Figure 3:**
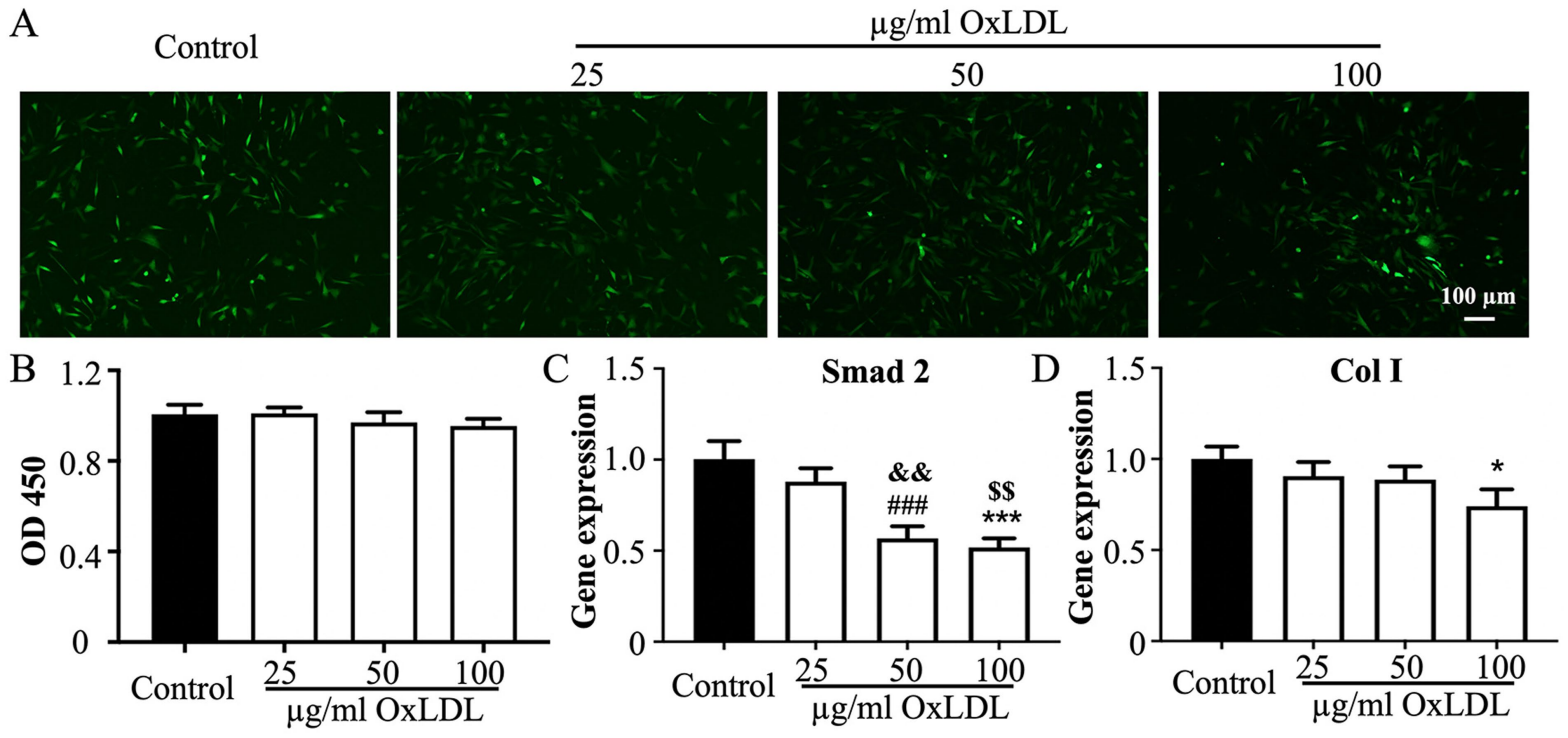
The effects of OxLDL on tenocytes. **(A)** Live/dead staining results in different group; **(B)** CCK8 results in different group; **(C,D)** the expression of tendon-related gene in different group (control group vs. 100 μ g/ml OxLDL group: *p < 0.05, ***p < 0.001; ontrol group vs. 100 μ g/ml OxLDL group: ###*p* < 0.001; 25 μ g/ml vs. 100 μ g/ml OxLDL group: ^$$^*p* < 0.005; 50 μ g/ml vs. 100 μ g/ml OxLDL group: ^&&^*p* < 0.005).

In order to further verify the effect of hyperlipidemia on tendon through cell experiments, we used different concentrations of OxLDL (25, 50 and 100 μ g/ml) to stimulate tendon cells. According to Live/Dead staining, the OxLDL did not affect the cell viability of tenocytes (Figure 3A and Supplementary Figure S3). Furthermore, the CCK8 analysis showed that although the cell proliferation ability decreased with the increase of OxLDL concentration, it was not statistically significant (Figure 3B). Similarly, flow cytometry results showed that different concentrations of oxLDL did not affect the cell proliferation cycle of tenocytes (Supplementary Figure S4). To examine whether hyperlipidemic state resulted in lower tendon-related gene expression, we tested the expression of three crucial transcription factors, including SCX, Collagen 1, and Smad2 genes. After 5 days culturing, the expression of SCX, Col 1a1, and Smad2 were suppressed by high concentration OxLDL (Figure 3C). Therefore, we conclude that OxLDL also altered the tendon-related genes.

### 3.4 The effects of hyperlipidemia on tendon repair process

**Figure 4:**
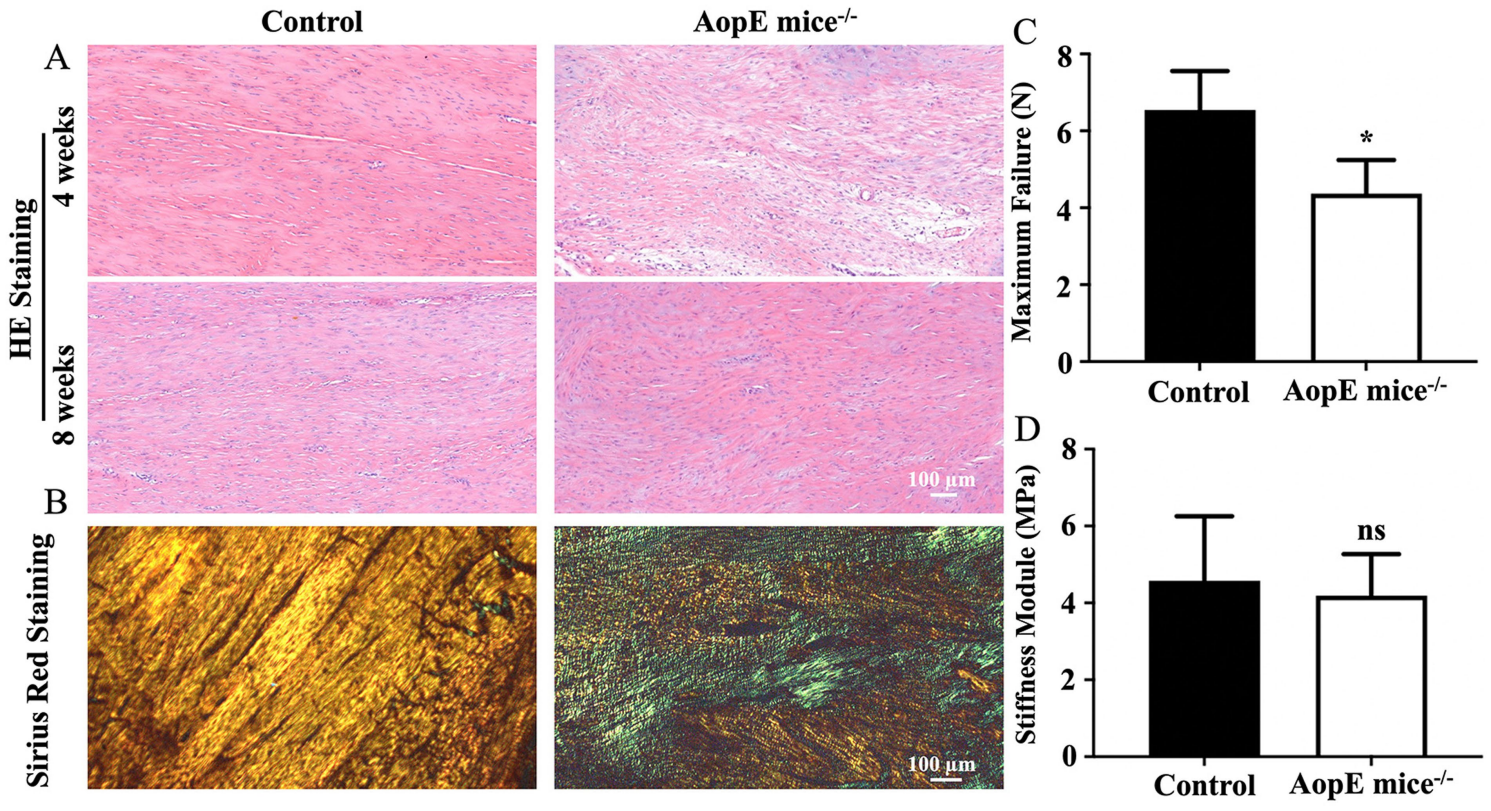
Evaluation of tendon repair performances in hyperlipidemic state *in vivo*. **(A)** H&E staining results in different group at postoperative 4 and 8 weeks; **(B)** The Sirius Red staining results in different group at postoperative 8 weeks; **(C,D)** The biomechanical results at postoperative 8 weeks (*p < 0.05).

Maintaining tissue function encompasses both physiological homeostasis and regenerative capacity following injury. To evaluate the repair performance, we established Achilles tendon injury models in control and ApoE−/− mice and performed histological evaluation at postoperative 4 and 8 weeks. According to the H&E staining results, the fiber structure of the repaired tendon tissue in high-fat mice was more disordered and the interfibrillar gaps were wider (Figure 4A). The sirius red staining results also showed that the fibers in the control group were brighter at postoperative 8 weeks, indicating a higher content of type 1 collagen and a more orderly arrangement of the fibers (Figure 4B). The related biomechanical results also demonstrated that the maximum failure stress of ApoE−/− group was significantly reduced compared to control group, and the elastic modulus was also reduced which was not statistically significant (Figures 4C,D).

## 4 Discussion

Hyperlipidemia induces metabolic and structural abnormalities in various tissues of the musculoskeletal system, leading to chronic injury. Some studies have reported an increased incidence of rotator cuff injury (RCI) with impaired healing capacity in hyperlipidemic patients (18, 19). In this study, we observed prominent collagen fiber disorganization and significant intracellular lipid deposition within tendon tissues of hyperlipidemic ApoE−/− mice. Further *in vitro* experiments revealed that exposure to the oxidized low-density lipoprotein (OxLDL) did not affect tendon cell proliferation or the cell cycle. However, OxLDL disrupted normal tendon metabolic homeostasis by suppressing the TGF-*β*/Smad2 signaling pathway. Moreover, injured tendon tissue exhibited poorer repair capacity compared to controls. Finally, our findings demonstrate that hyperlipidemic conditions disrupt the tendon homeostasis and impair tendon repair.

Although the present study did not identify an OxLDL concentration threshold that inhibits tenocyte proliferation at 100 μg/mL, the concentrations employed herein induced significant functional alterations in tenocytes. Consistent with previous studies, excessively high concentrations of oxidized low-density lipoprotein have been shown to markedly suppress endothelial cell proliferation (20, 21). Thus, we set the OxLDL concentration range at 25–100 μg/mL to avoid non-specific tenocyte damage that would otherwise interfere with the experimental results. Mechanistically, *in vitro* evidence demonstrates that OxLDL suppresses TGF-*β* and lysyl oxidase (LOX) expression while activating matrix metalloproteinases (MMPs) in tendon cells (13). TGF-β stimulates collagen fiber synthesis, and LOX catalyzes collagen cross-linking and maturation—both critical for functional ECM formation. Conversely, MMP activation degrades collagen fibers. OxLDL-induced dysregulation of these pathways consequently disrupts tendon ECM synthesis and remodeling, directly compromising tissue mechanical and structural integrity (13). Supporting our findings, studies in ApoE^−/−^ mice reveal significantly reduced collagen density, increased intra- and extracellular lipid deposition, enlarged tendon cross-sectional area, and disorganized collagen microstructure (22). These observations align with and extend our histological results. Furthermore, hyperlipidemia alters the expression of key ECM components in tendons—including collagen types I, III, IV, V, and VI, and MMP2/MMP9—reinforcing our tissue-level findings (23). Notably, hyperglycemic conditions also increase interfibrillar gaps in murine tendons, linked to reduced Egr1 expression. Downregulation of Egr1 decreases TGF-β1 levels, inhibiting collagen synthesis and altering tendon microstructure (24, 25). While different from hyperglycemia, hyperlipidemia similarly disrupts collagen-associated pathways and induces collagen disorganization. Our *in vitro* data also showed the similar result, establishing that hyperlipidemic conditions dysregulate tendon tissue homeostasis.

Related research focused on hyperlipidemia-induced tendon injury involves not only defective collagen synthesis but also direct tissue damage. Studies indicate that under hyperlipidemic conditions, OxLDL upregulates inflammatory cytokines and chemokines while promoting matrix metalloproteinase (MMP) activation. This cascade accelerates tendon degradation and impairs tissue homeostasis (5). OxLDL overload within peritendinous macrophages leads to pathological lipid accumulation and foam cell formation in the tendons of familial hypercholesterolemia (FH) patients. This process elevates pro-inflammatory factors (e.g., TNF-*α*, IL-8, IL-6), resulting in excessive inflammation that culminates in structural damage and tendon xanthoma (TX) development (21). Additionally, OxLDL generates reactive oxygen species (ROS), directly compromising rotator cuff (RC) integrity—a key molecular mechanism underlying tendon injury (23). OxLDL disrupts lipid packing within cells, altering membrane biomechanical properties. This impairs membrane repair and reduces cell migration efficiency, contributing to localized tissue damage (26). Thus, our results uncover complementary mechanisms contributing to OxLDL-induced tendon injury. Contrasting with tendon pathology, cardiovascular research demonstrates that OxLDL: (i) Enhances cardiac fibroblast proliferation, triggering increased synthesis of collagen type I and extra domain A (EDA)-containing fibronectin, and stimulates collagen I secretion—ultimately promoting myocardial fibrosis (27); (ii) Via its component lysophosphatidylcholine (LysoPC), upregulates biglycan and collagen I production in human aortic valve interstitial cells (AVICs), driving valvular calcification (28). Notably, tenocytes and cardiomyocytes differ markedly in oxygen consumption, metabolic profiles, and regenerative capacity. These fundamental distinctions suggest cell-type-specific responses to OxLDL exposure.

The *in vitro* results demonstrate that hyperlipidemia suppresses the TGF-*β*-Smad2 signaling pathway. This suppression inhibits the synthesis of the tendon-specific protein Scleraxis (Scx) and collagen, ultimately leading to tendon tissue disrupt. The Smad family members are key intracellular signaling mediators for the TGF-β superfamily. Under TGF-β stimulation, Smad2/3 accumulates within the nucleus, forms heteromeric complexes and undergoes nuclear translocation, which would transduce TGF-β signals (29). In tendon tissue, the TGF-β-Smad2 pathway induces expression of the tendon-specific transcription factor Scleraxis, which directly binds to promoter regions of type I collagen, including Col1a1and Col1a2, and type III collagen (Col3a1) genes (12). Some studies demonstrate that exosome-mediated activation of Smad2/3 signaling promotes tenocyte proliferation and differentiation while suppressing early inflammation, thereby enhancing tendon healing *in vivo* (30). Similarly, Matos et al. engineered remotely magnet-responsive functionalized magnetic nanoparticles (MNPs) that activate Smad2/3. This activation enhanced Smad2/3 phosphorylation and nuclear co-localization, stimulating the expression of tendon-related genes and deposition of tendon-associated proteins to facilitate tissue repair (31). While Smad2/3 signaling is essential, its activation via phosphorylation is critical for biological function. For instance, blocking Smad2/3 phosphorylation at Ser213 in an osteoarthritis model inhibited the protective effects of TGF-*β* signaling on hypertrophic chondrocytes, exacerbating disease progression (32). Furthermore, in the context of dyslipidemia-induced cardiovascular disease, OxLDL suppresses both total Smad2/3 and phosphorylated Smad2/3 (p-Smad2/3) expression in endothelial cells (33, 34). Significantly, our study provides the first evidence that hyperlipidemia reduces the expression of both total Smad2/3 and its functional phosphorylated form (p-Smad2/3) in tendon tissue. This dual suppression, which affected both overall protein levels and the activated fraction essential for signaling, impairs tendon tissue homeostasis. These findings reveal a previously unrecognized mechanism by which oxLDL disrupts tendon biology and identify the TGF-*β*-Smad2/3 pathway as a novel target of oxLDL in tendon tissue.

To sum up, hyperlipidemia disrupts tendon homeostasis by suppressing the TGF-β/Smad2 signaling axis via oxLDL, leading to diminished expression of tendon-specific markers (e.g., Scleraxis/Scx) and collagen synthesis. *In vivo*, hyperlipidemic ApoE−/− mice displayed disorganized tendon collagen with fibrotic gaps and lipid deposits, paralleling *in vitro* findings where oxLDL impaired TGF-*β*/Smad2-dependent ECM remodeling, a critical enzyme for collagen crosslinking. Mechanistically, oxLDL not only decreased total Smad2/3 levels but also inhibited their phosphorylation (p-Smad2/3), essential for nuclear translocation and transcriptional activation of tendon-repair genes. These molecular aberrations align with clinical observations of poor tendon healing in hyperlipidemic patients. Targeted reactivation of TGF-*β*/Smad2 signaling, such as nanoparticle-mediated Smad2/3 activation or exosome-based therapies, could restore tendon repair capacity in hyperlipidemia. Future studies should clarify tissue-specific oxLDL effects on Smad2/3 phosphorylation dynamics and explore interventions to enhance collagen synthesis and crosslinking, potentially mitigating hyperlipidemia-induced tendon degeneration.

This study still has several limitations and shortcomings that warrant further in-depth investigation in future research. First, while the inhibitory effect of hyperlipidemia on the TGF-*β*-Smad2/3 pathway was verified at the protein expression level in this study, and the validation experiments were conducted around the well-characterized canonical pathway with existing evidence sufficient to elucidate the core mechanism, whole-transcriptome sequencing and systematic high-throughput bioinformatics analyses were not performed. However, focusing solely on the TGF-β/Smad2 signaling pathway and its associated molecules precludes the analysis of potential key genes and regulatory networks underlying the physiological and pathological changes in tendon tissue regulated by hyperlipidemia and OxLDL at the genome-wide level, which to a certain extent limits the depth and breadth of the present study. In addition, constrained by the technical challenges associated with the isolation of tendon stem/progenitor cells (TSPCs), only mature tenocytes were used in the experimental assays of this study. As the core cell population with proliferative and differentiative potential in tendon tissue, TSPCs may exhibit distinct biological behaviors and regulatory mechanisms from mature tenocytes in response to hyperlipidemia and OxLDL stimulation. In future research, we will further supplement experiments with multiple concentration gradients, perform whole-transcriptome sequencing and bioinformatics analyses to deeply explore the molecular mechanisms and cellular regulatory networks underlying hyperlipidemia-induced tendon degeneration. Meanwhile, we will further clarify the tissue-specific effects of OxLDL on the dynamic phosphorylation of Smad2/3 in different tissues and explore interventional strategies that can enhance collagen synthesis and cross-linking. These efforts aim to provide novel insights for alleviating hyperlipidemia-induced tendon degeneration and facilitate its clinical translation.

## Data Availability

The original contributions presented in the study are included in the article/Supplementary material, further inquiries can be directed to the corresponding authors.
