# Supplementary material for: Hyperlipidemia-induced metabolic dysregulation impairs tendon homeostasis: the role of TGF-β/Smad2 signaling pathway in lipid-mediated extracellular matrix remodeling
Source: Front Med (Lausanne). 2026 Apr 17;13:1820393. doi: 10.3389/fmed.2026.1820393 (PMC13132780; doi:10.3389/fmed.2026.1820393)
Supplement: Supplementary file 1 [file Data_Sheet_1.PDF]

Supplementary Material

1 Supplementary Figure

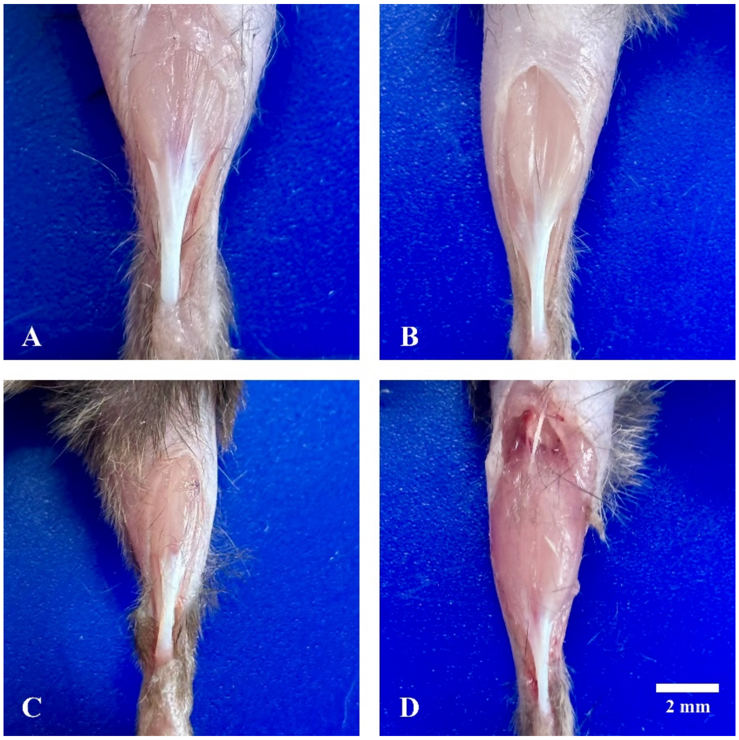

Supplementary Figure 1 Gross morphology of collected tendon samples: Figure A and B show tendons from the control group, while Figure C and D show tendons from ApoE-/-.

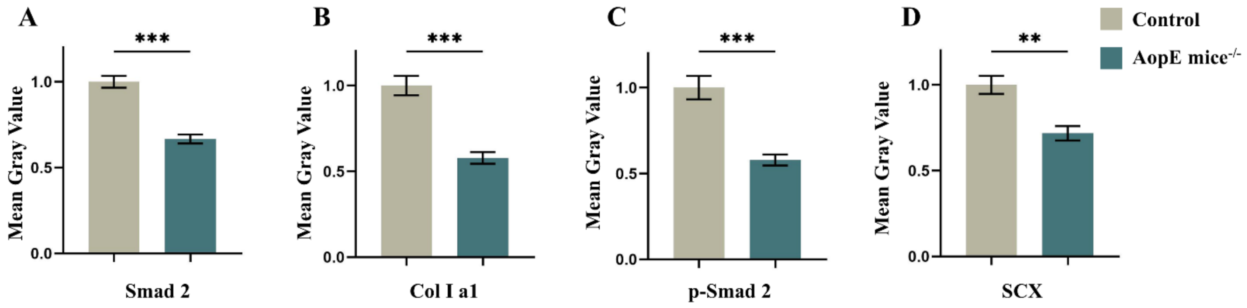

Supplementary Figure 2 Western blot detection and quantitative analysis results: Quantitative analysis of protein expression levels of Smad 2, Col I a1, p-Smad 2 and SCX (A-D) in tendons from control and ApoE-/- mice. (***P<0.005, ***P<0.001)

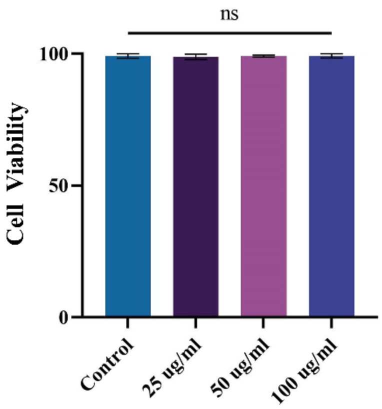

Supplementary Figure 3 Live/Dead staining was performed to evaluate cell viability: No significant differences were observed between the 25, 50 and 100 µg/ml OxLDL and the control group.

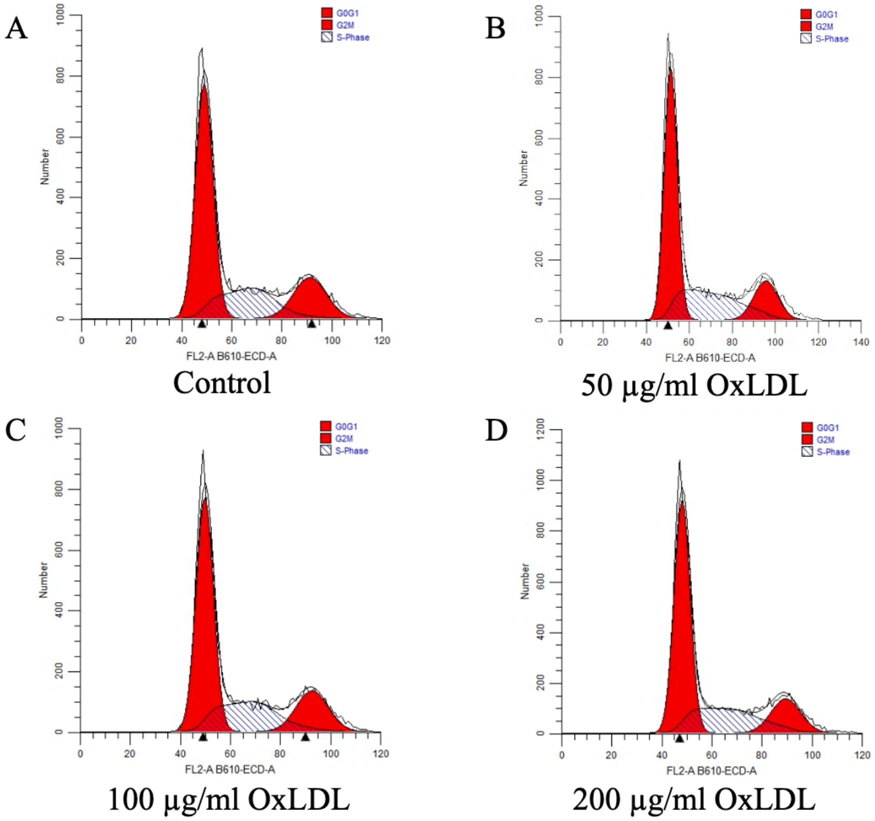

Supplementary Figure 4 The flow cytometry of cell cycle in different groups: (A) Control and (B-D) 50, 100, 200 µg/ml OxLDL.
